# Development and validation of an interprofessional collaborative educational module on the self-management of foot for individuals with type II diabetes mellitus

**DOI:** 10.12688/f1000research.146943.2

**Published:** 2024-09-06

**Authors:** Sushma Prabhath, Harihara Prakash Ramanathan, M Ganesh Kamath, Gagana Karkada, Ganesh Handady, Ciraj Ali Mohammed, Arun G. Maiya

**Affiliations:** 1 Departments of Anatomy and Medical Education, Kasturba Medical College Manipal, Manipal Academy of Higher Education, Manipal, Karnataka, India; 2 MAHE-FAIMER Institute for Leadership in Interprofessional Education, Manipal Academy of Higher Education, Manipal, Karnataka, India; 3 Department of Physiotherapy, KM Patel Institute of Physiotherapy, Pramukhswami Medical College Campus, Bhaikaka University, Karamsad, Gujarat, India; 4 Department of Physiology, Manipal University College Malaysia (MUCM), Bukit Baru, Melaka, Malaysia; 5 Centre for Diabetic Foot Care and Research (CDFCR), Department of Physiotherapy, Manipal College of Health Professions, Manipal Academy of Higher Education, Manipal, Karnataka, India; 6 Department of Pediatrics, Kasturba Medical College Mangaluru, Manipal Academy of Higher Education, Manipal, Karnataka, India; 7 Department of Medical Education, College of Medicine and Health Sciences, National University of Science & Technology, Al Athaiba, Muscat Governorate, Oman

**Keywords:** Interprofessional, collaborative, educational module, diabetic foot self-management, development, validation

## Abstract

**Background:**

Insufficient awareness of foot self-care among diabetic individuals results in diabetic foot ulcers. The management of diabetes and diabetic foot ulcers demands a well-coordinated approach that involves multiple healthcare providers (HCPs). The present study aims to develop and validate an interprofessional collaborative (IPC) educational program involving HCPs to efficiently oversee and instruct the public on appropriate strategies for self-managing diabetic foot health.

**Methods:**

The research group worked on creating an educational module titled ‘An Interprofessional Collaborative Educational Module on Self-Management of Foot for Individuals with Type II Diabetes Mellitus.’ The objective of this module was to promote the adoption of proper practices in self-managing foot health for individuals with type 2 diabetes mellitus. A panel of 13 experts participated in a two-stage validation process using the Delphi method to assess the module and its educational resources. Subsequently, the module was tested on a group of 30 participants, *i.e.*, individuals with diabetes, with its efficacy evaluated through conversation analysis and in-depth interviews.

**Results:**

The three-month-long module included three sessions

• 1: Diabetes and its health implications
• 2: Diabetic foot and self-management
• 3: Interprofessional education in diabetic foot care

The mode of content delivery was via Whatsapp, and the educational resources, in the form of pamphlets, flowcharts, handouts, case-based cartoons, and videos on diabetes, including diabetic foot, its risks, and self-management, were shared regularly.

All participating experts consensually validated the module and educational resources. Analysis of in-depth interviews revealed that the module immensely benefitted the participants and helped them improve their knowledge and practices of foot care in diabetes.

**Conclusions:**

The study concludes that IPC educational modules can enhance adherence to proper diabetic foot care practices, potentially reducing the occurrence of foot ulcers and amputations, and ultimately improving the quality of life for individuals with diabetes.

## Introduction

Foot ulcers represent persistent issues that arise as a result of diabetes. People with diabetes face a significant lifetime risk of developing a foot ulcer, with a chance of up to 25%. Alarmingly, it is estimated that every half a minute, a lower limb is lost globally due to diabetes-related complications. ^1^

Diabetic foot ulcers (DFUs), if severe, may lead to lower limb amputations and even death. As a result it can thus disrupt the quality of life for the person involved and their family. ^2^

In 2017, the worldwide prevalence of diabetes mellitus impacted almost 425 million individuals, and it is projected to increase to 629 million by the end of 2045. ^3^ In India, over 77 million people currently have diabetes, and this number is expected to surge to 35.7 million by 2045. Diabetes affects approximately 8.9% of the Indian population and is associated with an estimated one million deaths annually. ^4^^,^^5^

The worldwide occurrence of DFUs varies between 5% and 7.5% among individuals with diabetic neuropathy. ^6^ In India, a survey conducted within the community found the prevalence of diabetic foot ulcers to be 6.38%. ^7^ Approximately 15% of DFUs probably deteriorate, ultimately resulting in lower extremity amputations, a problem that poses significant global clinical management challenges and places a substantial economic burden on society. ^7^

Research findings indicate that around 25% of people with diabetes in India are anticipated to experience DFUs. ^8^^,^^9^ This situation could be exacerbated by insufficient general awareness, limited medical facilities, and economic constraints. ^10^^,^^11^ India’s population comprises various ethnic and genetic groups with distinct cultural beliefs and hygiene practices. ^12^ This diversity can significantly impact the causes of diabetes, its outcomes, such as diabetic foot ulcers, and individuals’ responses to diabetes treatments. ^4^^,^^13^

Diabetic care-related practices may also vary based on ethnicity, lifestyle practices, socio-economic status, and the prevalence of diabetes. It may further contribute to an increase in the occurrence of DFUs in India. ^14^^,^^15^

Research has revealed that individuals with diabetes frequently overlook self-care measures for their feet and tend to implement foot-care practices only after complications have already developed. ^1^

Insufficient awareness about diabetes-related foot issues and self-care practices among diabetes patients stands as a significant contributor to the development of diabetic foot ulcers. Inadequate control of blood sugar levels, non-compliance with dietary guidelines, and a lack of exercise exacerbate this issue. Inadequate knowledge and poor foot care practices are noteworthy risk factors for foot complications in diabetes. ^16^

Surveys in Indian populations have also revealed an evident gap between foot care knowledge and practices among diabetic individuals. ^15^^,^^17^^,^^18^ These studies have further highlighted the need for increasing awareness about proper foot care practice in diabetic patients to reduce the incidence of complications. They added that this can be achieved by educating individuals with diabetes about self-foot care and other essential practices. ^17^^,^^18^

Implementing an educational program focused on self-managing diabetic foot issues could be a viable solution. ^19^

Educating people about self-management of diabetic foot conditions has enhanced foot care practices, lowering the chances of developing diabetic foot ulcers. ^20^^,^^21^ Individual patient education of five to six minutes helped improve foot care practices, according to reports from the diabetic rural population of Puducherry, India. ^22^ The study added that such educative sessions, when consistently reinforced, are likely to result in healthy habit formation, prevent disabilities, and reduce medical expenses in the long run. ^22^ However, there is a paucity in the available literature regarding such attempts, *i.e.*, prolonged and continuous educational interventions on self-management of diabetic foot in the Indian scenario.

Therefore, there is an urgent necessity to introduce nationwide diabetes foot self-management educational initiatives in India. The primary objective is to raise awareness about preventing diabetic foot-related issues and enhance the management of diabetic foot care. These efforts have the potential to mitigate the frequency of foot amputations and alleviate the burden of ‘diabetic foot’ across the nation. ^23^

Diabetic foot self-management practices must be adequately and effectively communicated to individuals with diabetes. The complexity of this disease is such that it needs a collaborative approach involving various healthcare providers (HCPs) to achieve desired outcomes. This concern could be addressed through an interprofessional (IP) team-based approach.

An IP team of HCPs, such as physicians, endocrinologists, surgeons, physiotherapists (including an orthotist, and podiatrist), nursing faculty, and nutritionists can collaboratively focus on comprehensive diabetic foot care and its management. ^24^^,^^25^ An IP team-based approach is recommended as the most effective method for educating the population about the correct techniques of diabetic foot self-management. ^24^^,^^25^ Engaging an IP team in designing the educational intervention to disseminate optimal practices for diabetic foot self-management is likely to yield positive results. However, in countries like India, where a physician-centered or multidisciplinary approach to patient care is more prevalent, the efficacy of the IP team-based approach has yet to be extensively investigated.

Engaging HCPs and creating an interprofessional collaborative educational module focusing on the proper methods of diabetic foot self-management while ensuring its accessibility to individuals with diabetes could be a successful strategy. This approach will facilitate comprehensive and ongoing education, encompassing all critical aspects of diabetic foot self-management. As a result, it has the potential to decrease diabetic foot issues and their associated complications in people with diabetes, ultimately contributing to an enhanced quality of life for these individuals.

This current study aims to create and verify an interprofessional collaborative (IPC) educational module with the involvement of HCPs. This module is intended to efficiently instruct the population on appropriate strategies for self-managing diabetic foot issues.

## Methods

The study has been designed in adherence to the Declaration of Helsinki and has received approval from the Institutional Ethics Committee of Kasturba Medical College and Kasturba Hospital (IEC No 698-2020 dated Wednesday, 11 November 2020). It was additionally registered in the Clinical Trials Registry of India ( CTRI/2021/03/031629 dated Monday, 01 March 2021). Written informed consent has been obtained from the participants recruited for the study. The study was conducted between April 2021 and November 2022.

Reporting guidelines used:

TIDieR (Template for Intervention Description and Replication) for reporting the description of the educational intervention and its replication.

COREQ (COnsolidated criteria for REporting Qualitative research) Checklist for reporting the qualitative research carried out.

CONSORT (Consolidated Standards of Reporting Trials) checklist for reporting the usefulness of the educational intervention carried out as a clinical trial on the study participants, *i.e.*, individuals with diabetes.

**Figure 1.**
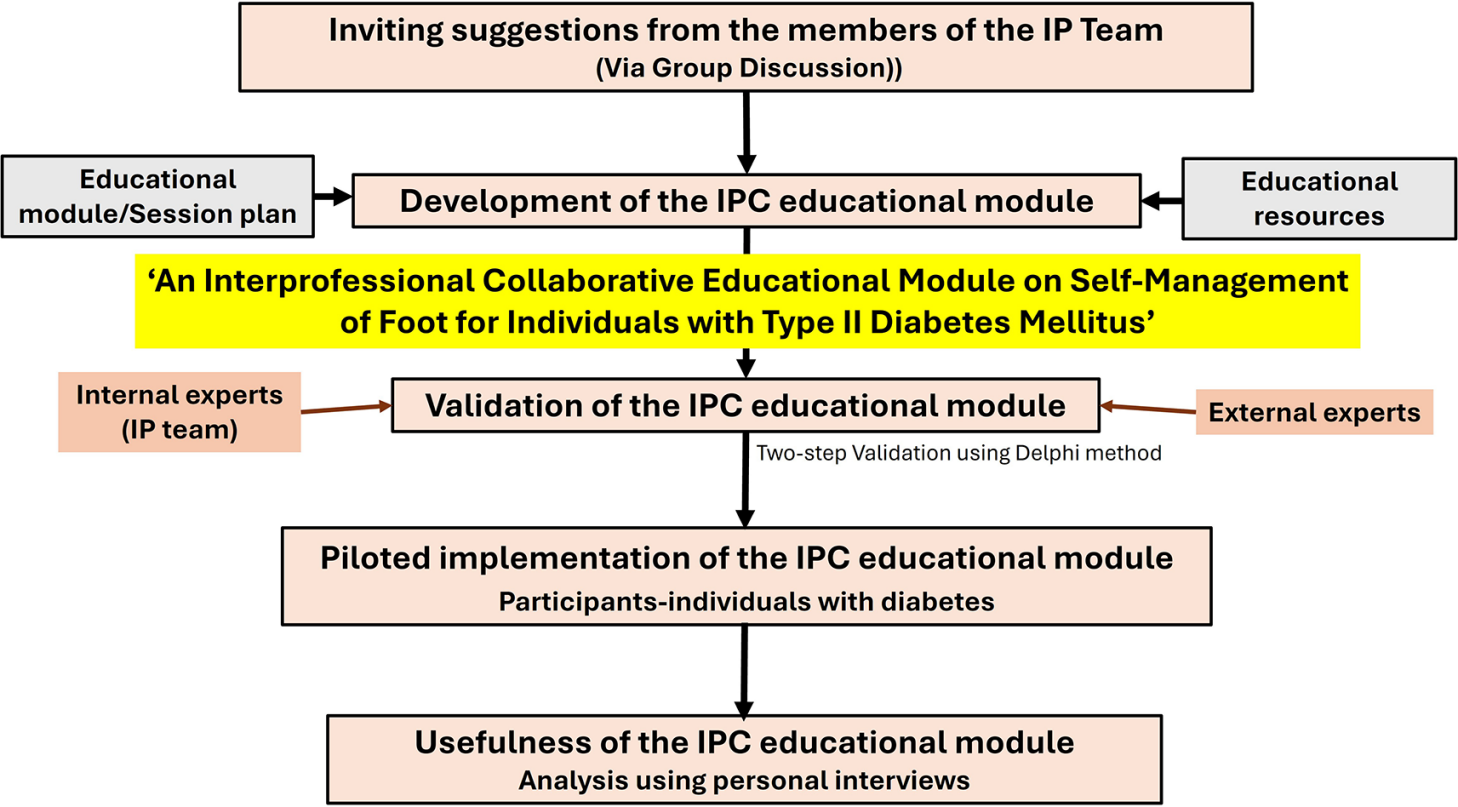
Methodology summarized as a flowchart.

A flowchart summarizing the methodology is presented in Figure 1.

### Development of the IPC educational module

The principal investigator (first author of the study) identified and invited the following HCPs, *i.e.*, a general physician, an endocrinologist, a surgeon, a physiotherapist, a dietician, and a nursing professional to constitute an IP team. The IP team also included an anatomist with experience in health professions education. All the HCPs invited to form the IP team were involved in diabetic foot care and management in their respective workplaces.

A group discussion (GD) was arranged for the members of the IP team to discuss the development of the educational module.

The IP team was previously constituted after seeking the perceptions of the HCPs related to the importance of interprofessional collaboration in teaching diabetic foot self-management. ^24^ The HCPs were aware of the benefits of an IP team-based approach in diabetic foot care. They further expressed their willingness to work as part of an IP team and suggested appropriate teaching methods for diabetic foot self-management. ^24^ This study thereby led to the conduct of the present study, *i.e.*, the development and validation of an interprofessional collaborative educational module on the self-management of foot for individuals with type II diabetes mellitus.

During the current study, the GD was conducted online via Microsoft Teams (Microsoft Corporation, United States). Each IP team member provided insights for the development of the interprofessional collaborative educational module.

The educational module titled “An Interprofessional Collaborative Educational Module on Self-Management of Foot for Individuals with Type II Diabetes Mellitus” was then developed by the research team involving the following components:

• Mode of delivery
• Level of learner (study population)
• Duration of the session
• Domains of learning
• Level of proficiency expected
• Educational resources
• Assessment and evaluation methods

The educational resources were developed after reviewing the literature on the following databases: Medline, Johns Hopkins Medicine, World Diabetes Foundation, Healthline, World Health Organization (WHO), Joslin Diabetes Centre, WebMD, National Institute of Health-National Institute of Diabetes and Digestive Kidney Diseases, and Google. The educational resources were developed in English and the local language, *i.e.*, Kannada.

The educational resources were initially developed in English and later translated into the local language, *i.e.*, Kannada. To ensure the consistency and accuracy of translated content, the following measures were taken:

*Back-Translation:* After translating the content from English to the local language, *i.e.*, Kannada, we had a different translator (who was not involved in the initial translation) translate the content back into English. It ensured that the translated version’s accuracy and consistency reflected the original content.

*Bilingual Expert Review:* We also invited bilingual experts (fluent in both English and Kannada) to review the translated content. The experts assessed the accuracy of the translation, ensuring that it captured both the technical and cultural nuances of the original material.

### Validation of the IPC educational module

*Identification of validators/review experts*

A total of 11 validators (six internal and five external) were involved in validating the educational module and resources.

The individuals who carried out the validation of the educational module were a team of both external and internal experts. The internal experts were the same HCPs who constituted the IP team and were previously involved in the FGD leading to the development of the educational module.

The external experts involved in the educational module validation were other HCPs as well as experts in the field of medical education who were unrelated to the study. They were not part of the IP team or the educational module’s development.

### The validation process

Two-step validation of the IPC educational module and resources was carried out using the Delphi method. ^26^

The first consensus stage was obtained using a validation tool (research instrument) adapted from Ribeiro and Spadella, 2018 ^27^ [Annexure 1 in extended data]. Appropriate permissions were obtained for its modification and usage in the current context.

The validation tool had two parts.

Part 1: Validation tool for the educational module

Part 2: Validation tool for educational resources

Both parts included the basic details of the validators, *i.e.*, age, gender, and years of professional experience.

Part 1 of the validation tool assessed domains-concept, didactic-pedagogical, operational, and adhesion features. Part 2 of the validation tool assessed the following components of the educational resources developed: concept, language, illustration, layout, and motivation ^40^ [Annexure 1 in extended data].

The evaluation tool was administered online via Microsoft Forms (Microsoft Corporation, United States).

The experts examined the assessment/dimension components, giving ratings ranging from zero to 10, where zero indicated complete disagreement and 10 signified total agreement. Each segment included three open-ended questions to gather suggestions for additions, revisions, or removals and feedback regarding errors and misunderstandings.

The assessments conducted by experts were examined, and the scores were recorded in a Microsoft Excel spreadsheet (Microsoft, Corporation, USA). This process included determining the mean value for each item to evaluate its perceived significance or suitability and calculating standard deviations to assess the degree of agreement among the experts. The established cut-off values were as follows: items with a mean ≥7 were considered important or adequate, while items with a mean <7 were considered minor or appropriate. Additionally, items with a standard deviation of <3 were considered to be in consensus, whereas those with a standard deviation ≥3 were deemed non-consensual. Subsequently, this data matrix served as a guide for refining the initial versions. ^27^ The validated items had close-to-cut-off results and increased the importance level.

After incorporating the required suggestions and making the necessary improvements and inclusions, the module and resources were again shared with the validators for review, and the second stage of consensus was obtained.

### Piloted implementation of the IPC educational module

After approval and validation, the implementation of the educational module was planned with the consensus of the members of the IP team.

*Study population*

The study population included individuals with Type II Diabetes Mellitus.

*Inclusion and exclusion criteria*

Inclusion criteria

• The individuals with Type II Diabetes Mellitus for any duration were considered. The study participants were recruited from the Centre for Diabetic Foot Care and Research (CDFCR) Department of Physiotherapy, Kasturba Hospital Manipal.
• Participants who were comfortable using the social media tool “ WhatsApp”, were preferred.

Exclusion criteria

• Patients with type I diabetes, impaired fasting glucose, impaired glucose tolerance, and gestational diabetes were excluded from the study.
• Patients who had diabetic foot ulcers were also not considered.

The sample size calculation was calculated out obtain a small effect (change). The calculation was done with G*power 3.1.9.4 software for sample size calculation based on t tests - Means: The difference between two dependent means (matched pairs).

*Sample size calculation*

$$\underset{}{\text{Small effect}}:265+10\%\left(\text{Loss to follow-up}\right):265+26=291\;\text{participants}$$

To check its effectiveness, the module was initially implemented only to a pilot (small) group of study participants (N=30), *i.e.*, individuals with Type II Diabetes Mellitus. Participants were recruited after explaining the entire process of educational intervention and obtaining written informed consent.

The principal investigator created a WhatsApp group for the study participants and educational resources were shared there. The information regarding creating the WhatsApp group and sharing educational resources was previously conveyed to the participants during the recruitment process. The participants were encouraged to review the resources on a regular basis. To ensure whether participants read or watched the resources shared on WhatsApp, the number of views per entry was reviewed and tracked. No other measures were taken to ensure participants’ fidelity/adherence to the intervention. The users (participants) used their own internet data pack to access WhatsApp. No internet data bundle facilities or incentives were provided to the participants for the same by the researchers.

**Figure 2.**
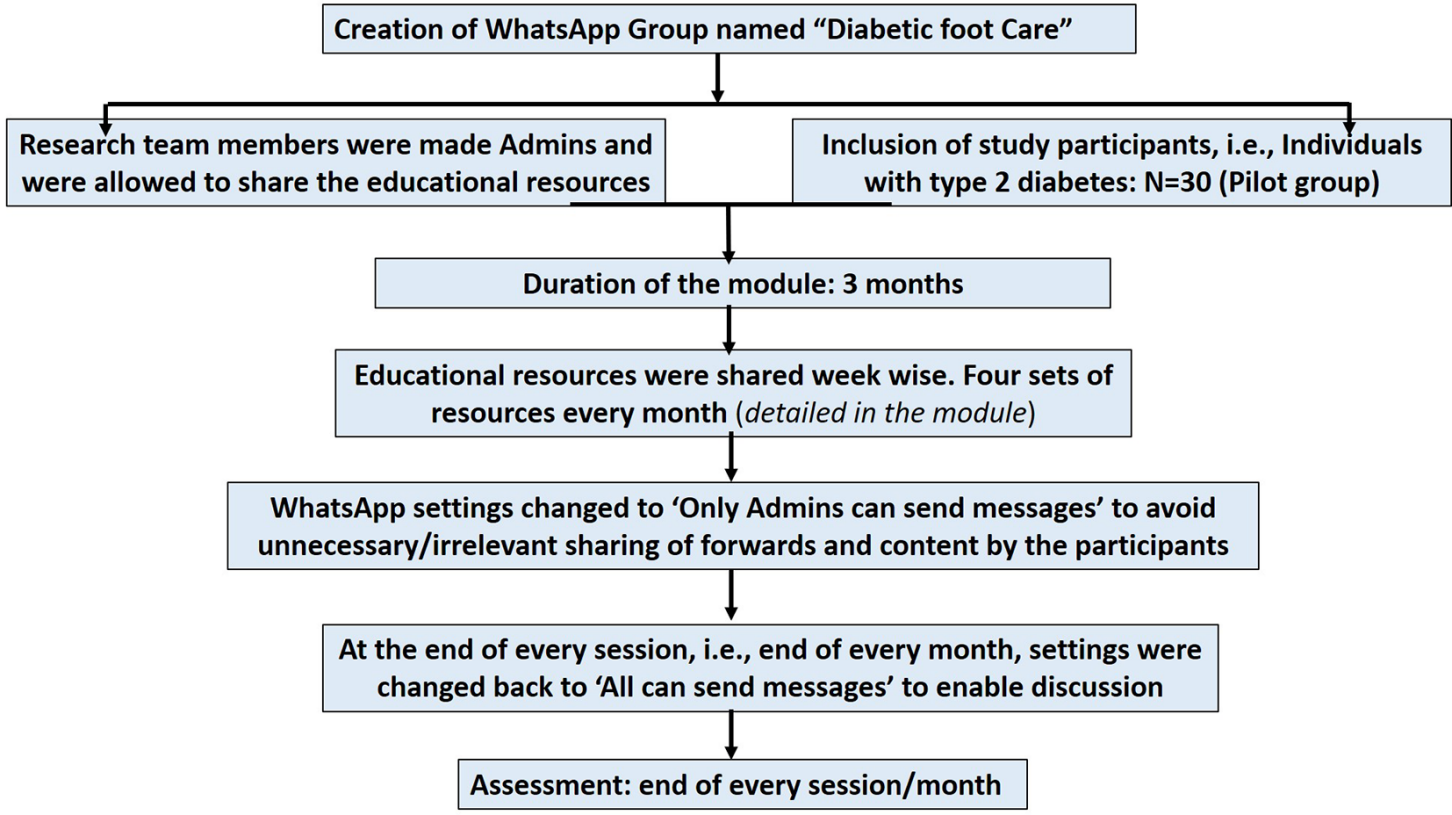
A flow chart showing the implementation steps of the interprofessional collaborative (IPC) educational module on diabetic foot care.

A flow chart showing the implementation steps of the IPC educational module is represented in Figure 2.

Feedback was sought from the study participants regarding the usefulness of the educational module by inviting them to express their views over the chat.

The statement read this way:

*“Hope you like the way in which the session is being conducted. Hope the resources that are shared here are useful. We have changed the settings for you to reply.*

*Kindly give a 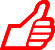 or 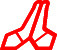 or type YES if this session is useful to you. You may also add your suggestions (if any) for further improvement of the session. You can also send a voice message.”*

The settings that were initially changed to “only admins can edit the group info” while sharing the educational resources were changed to “all participants can send messages to this group” enabling the participants to express their views.

The module’s effectiveness was further analyzed by inviting the study participants to a questionnaire-based telephonic interview with the principal investigator (first author of the study). A convenient sampling approach was used for this purpose.

At the end of the implementation of the education module, the participants were asked to express their willingness (in the WhatsApp group) to attend a telephonic interview related to the usefulness of the session. The consenting participants were later telephonically approached for the interview.

The interview guide used for this purpose was self-developed and validated by five medical and IP education experts. The interview guide is included as Annexure 2 in the extended data. ^40^ Each interview lasted for 45-60 minutes and was audio recorded. Field notes were also made during the interview by the interviewer. The interviewees’ responses were subjected to thematic analysis, and themes were identified.

### Data analysis

The interview recordings were transcribed to text, and the final transcripts were verified for clarity and correctness by members of the study team. Participants in the research were categorized as participants 1, 2, 3, and so on to guarantee anonymity and de-identification. The transcripts were then imported for processing using MAXQDA Analytics Pro 2020 (VERBI GmbH, Berlin, Germany) The free trial version of the software was used.

### Thematic analysis

Thematic analysis (content analysis) was performed on the participant replies. The authors (three of them) coded the material after reading and rereading the participant replies. The codes were then examined to find the pattern, and the themes were identified. To identify the topics, an inductive and semantic technique was used.

The authors created an initial coding scheme with the first two transcripts, which was enhanced by examining two more transcripts. The codebook was improved further iteratively with consecutive transcripts until consensus was attained. The coding was reviewed within and between transcripts.

The entire research team approved the final codebook, which was applied to all transcripts. Following coding, comments were evaluated for frequency by topic matter.

## Results

### Development of the IPC educational module

Considering the input provided by the members of the IP team, the research team dedicated their efforts to creating an IPC module titled “An Interprofessional Collaborative (IPC) Educational Module on Self-Managing Foot Health in Individuals with Type II Diabetes Mellitus.”

The objective of the module was to instill self-management practices for foot care in individuals with Type II Diabetes Mellitus.

The module was designed to run for three months, consisting of three sessions.

• 1: Diabetes and its Health Implications
• 2: Diabetic Foot and Self-Management
• 3: Interprofessional Education in Diabetic Foot Care

Considering the ease of accessibility, the IP team unanimously suggested that the mode of delivery should be online. It was decided that the resources would be shared regularly through WhatsApp.

The learning outcomes, domains of learning involved, level of proficiency expected, educational resources, assessment methods, and evaluation were clearly defined for each session (as detailed in the educational module- Annexure 3 in extended data ^40^).

The educational resources were mainly of the following types (Annexure 4 in extended data ^40^):

• Educational materials include pamphlets, flowcharts, handouts, case scenarios, and cartoons addressing diabetes and its health consequences, focusing on diabetic foot issues, associated risks, and self-management.
• Insights from leading experts in the field of diabetic foot care, emphasizing the advantages of sound foot care practices, presented as “What do the experts say?”
• Informative videos on the correct methods for diabetic foot care.
• Newspaper articles discussing diabetic foot care practices, authored and published in regional newspapers.
• Group discussions involving the study participants centered around self-management of diabetic foot health.

The effectiveness of the entire module was evaluated using conversation analysis and in-depth interviews.

### Validation of the IPC educational module

A total of 11 experts validated the module. Out of them, five were internal experts, *i.e.*, members of the IP team, and six were external validators. The average age of the validators was 44 years, ranging from 35 to 60 years. Their average professional experience duration was 19 years, spanning from 10 to 35 years.

The data matrix generated in this phase displayed significant means and standard deviations for every item assessed in the educational module and resources. On average, the scores for each item were 8.5 or higher. Furthermore, all items aimed to secure consensus among experts, with standard deviations remaining below 1.5 (as indicated in Tables 1 and 2).

**Table 1.** Mean and standard deviation for items assessed by experts in the interprofessional collaborative (IPC) educational module.

Items to be assessed (Mean±SD)	Session 1	Session 2	Session 3
**1. Concept domain**			
1.1 The themes and content proposed are relevant for establishing the practices of Self-Management of foot among patients with Type II Diabetes Mellitus	8.6±0.69	9.2±0.91	9.3±0.67
1.2 The themes and content proposed are adequate for the target audience (individuals with Type II Diabetes Mellitus)	8.8±0.78	8.9±0.73	9±0.81
1.3 The themes and content proposed are enough to supply the target audience’s needs	8.9±0.73	8.9±0.73	8.8±0.63
1.4 The themes and content proposed to allow cognitive ownership by the target audience about self-management of diabetic foot	8.9±0.73	8.7±0.67	8.6±0.51
1.5 The depth of themes proposed is suitable for the target audience	9.2±0.63	9.2±0.78	9.2±0.63
**2. Didactic/pedagogical domain**			
2.1 The program content of each module is clear and objective	9±1.24	9.3±0.67	9.3±0.67
2.2 There is coherence between the modules’ objectives and program content	9±1.05	9.1±0.73	9.2±0.63
2.3 The teaching strategies are suitable for the target audience	8.9±0.73	9.2±0.78	9.1±0.56
2.4 The learning activities proposed to allow autonomous learning	8.8±1.13	8.8±1.13	8.9±0.56
2.5 The support material proposed for use during activities favors understanding of the modules’ content	9±0.47	9.2±0.63	9.1±0.73
2.6 Didactic resources are easily understandable and foster learning by the target audience	9±0.66	9±0.66	8.9±0.73
2.7 References used are pertinent and representative	9.4±.69	9.3±0.67	9.1±0.56
2.8 Individual and collective evaluation process is adequate	8.9±0.56	9±0.66	9.1±0.56
**3. Operational domain**			
3.1 The execution timetable of modules is adequate	9.2±0.78	9.1±0.73	9±0.94
3.2 The mode in which activities of each module are conducted is adequate	9.3±0.67	9.2±0.63	9.2±0.63
**4. Adhesion domain**			
4.1 The educational strategy proposed will encourage and motivate the participation of the target audience	9.2±0.78	9.1±0.73	9±0.66
4.2 Activities proposed in modules allow frequent activity practice by the target audience	9±1.0	8.9±0.87	8.8±0.78

(0 - complete disagreement and 10 - total agreement).

**Table 2.** Mean and standard deviation for items assessed by experts in the educational resources to be used in the interprofessional collaborative (IPC) module.

Items to be assessed	Educational resources for Session 1	Educational resources for Session 2	Educational resources for Session 3
**1. Concept**			
1.1 The content covered is relevant for the promotion of the practices of Self-Management of foot among patients with Type II Diabetes Mellitus	8.78±0.83	8.89±0.92	9±0.5
1.2 The content is suitable for the target audience (individuals with Type II Diabetes Mellitus)	8.78±1.30	8.78±0.97	8.78±0.66
1.3 The content is enough to supply the target audience’s needs	8.33±0.70	8.78±0.97	8.67±0.70
1.4 The content can be easily applied to the target audience’s daily routine	8.4±1.33	8.78±0.83	8.78±1.09
**2. Language**			
2.1 Writing style is compatible with the target audience	8.67±1.58	8.78±0.97	9.1±1.39
2.2 Writing style is attractive	8.56±1.66	8.67±1.41	8.78±1.39
2.3 The language used is clear and objective	9±0.86	9.1±0.92	8.89±1.05
**3. Illustrations**			
3.1 Illustrations are adequate to and match the theme of the support material	8.89±1.05	8.78±1.30	8.56±1.13
3.2 Illustrations are clear and allow easy understanding	8.89±1.16	8.67±1.32	8.44±1.13
3.3 The number of illustrations is content-suitable in support materials	8.89±0.92	8.56±1.42	8.78±1.20
**4. Layout**			
4.1 The font type eases reading	8.67±1.22	8.89±1.05	9±1.11
4.2 Colors are adequate and facilitate reading	8.78±1.20	9±1.11	9±1.11
4.3 Visual composition is attractive and organized	8.89±1.26	8.89±1.26	9±1
4.4 The size (dimensions) and number of pages of the support material are appropriate	8.89±0.92	8.67±1.22	8.89±1.05
4.5 Content layout is adequate	9±0.86	8.67±1.41	8.78±1.20
4.6 Font size in headings and content is adequate	9±0.86	8.67±1.32	9±1.00
**5. Motivation**			
5.1 The content is motivating and encourages full reading	9.11±0.92	8.56±1.23	8.56±1.01
5.2 The content awakens the interest in of readers	8.78±1.30	8.78±1.30	8.89±1.05
5.3 The content solves doubts, clears things up, and educates the target audience	8.44±1.13	8.78±0.97	8.4±1.01

(0 - complete disagreement and 10 - total agreement).

The reviewers put forth the following thoughts and suggestions.

*For the educational module:*

*“As the topic is diabetic foot related better to focus on it from the first session. General discussion on diabetes and its impact on health can be restricted. Only normal sugar values and ideal control levels can be stressed. Stress that foot problems are neglected can affect the livelihood when everything else is normal”* ( **Expert 1)**

*“As the background educational level of the beneficiary is unknown, prepare the module applicable to illiterates also”* **(Expert 1)**

*“Comprehensively covers all aspects of foot care in diabetes”* ( **Expert 2)**

*For educational resources:*

*“As this topic is new to the target audience, if possible, we can add illustrations/images of how the IP team can come out to take care of the individual at the tertiary level. (just a suggestion, as it had more text than images/illustrations)”* ( **Expert 1)**

*“This resource provides an overview of how an interprofessional team works toward managing diabetic foot. It is simple and clear for a layperson to understand”* ( **Expert 6)**

The suggestions were incorporated, and necessary changes were made to the module and the educational resources.

### Piloted implementation of the IPC educational module

Subsequently, the IPC educational module was tested on a group of 30 willing participants who consented to participate in the study. The module was three months duration and was implemented from January to March 2022. An analysis of WhatsApp conversations conducted after the sessions concluded that the module had a substantial positive impact on the participants, enhancing their understanding and implementation of diabetic foot care practices.

*“The session was very informative and useful”* ( **WhatsApp conversation 1)**

*“Highly educative series. Learnt a lot about foot care and started following the tips given therein. Thanks for the valuable guidance given by your team”* ( **WhatsApp conversation 2)**

*“I sincerely THANK you people for providing excellent knowledge to us. The way you have given answers to the queries is adorable. I also look forward in other areas of health tips in future. God bless you!”* ( **WhatsApp conversation 3)**

**Figure 3.**
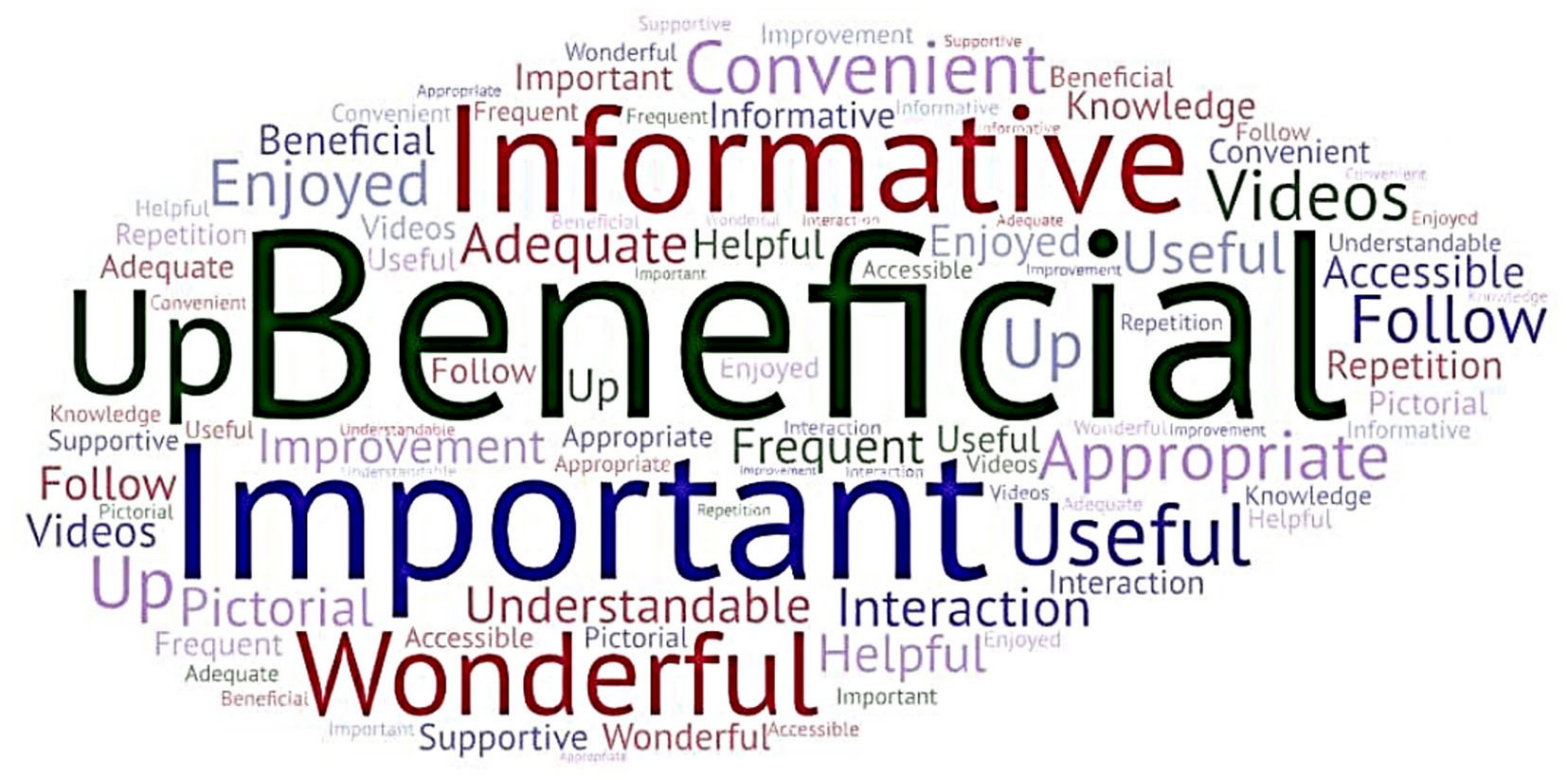
Word cloud (created at https://wordart.com/create) from the participants’ keyed responses on the usefulness of the interprofessional collaborative (IPC) educational module in diabetic foot care.

Among the 30 participants, seven agreed to a telephonic interview and shared valuable insights about the conduct of the IPC educational module. According to the interviewees, all sessions of the IPC educational module were beneficial. The sessions and shared information were highly relevant, appropriate, and easily understandable. As stated by the interviewees, it was a good learning experience that helped them follow and improve the proper foot care practices. Figure 3 depicts a Word Cloud created incorporating the participants’ keyed responses. The participants’ responses were subjected to thematic analysis, and themes were identified. The participants’ responses in the form of quotes and the themes derived are presented in Annexure 5 ^40^ in the extended data.

The major themes identified were: beneficial, appropriate, important, informative, useful, understandable, and improvement.

The observations made are presented below under the respective domains.

### The overall experience of being a part of the study

The participants stated that the overall experience of participating in the study was useful. In their view, it was helpful and was a good learning experience. It helped them learn and understand the proper foot care practice for diabetes.

The themes identified were beneficial, informative, and wonderful knowledge.

*“I got many benefits because aaa….. I am not aware of certain things, that you informed me, ahhh... so where the changes are required there I had made changes”* **(Participant 1)**

*“Based on our situation it was good and our foot should be cleaned and seeing about diabetes and diabetic foot, ummmm we get frightened. We should aaaah … maintain diet and our health ummm.. should be maintained, regarding food also, etc, we got an experience. Yes, it was useful”* **(Participant 4)**

*“It was wonderful, mmmm.. the three months program was really very helpful. I have gained lot of knowledge, which otherwise I was not knowing about diabetes”* **(Participant 5)**

### The usefulness of the sessions

The sessions were highly educative and useful, as stated by the study participants. The sessions helped them learn the proper practices of foot care in one setting rather than referring to multiple other sources, such as newspapers which the participants used to refer to gain information previously.

The themes identified were supportive and useful.

*It was very useful for me, ahhhh … like how we should maintain our health. Before we didn’t knew information regarding this, ummmm …..was reading through newspapers regarding diabetes. It had given us more information ummmm … like what should not be done. And it ummm … helped a lot.* **(Participant 4)**

### Type of learning materials that evoked interest in learning the proper foot care practices

The type of learning materials that mainly evoked the participants’ interest in learning the proper foot care practices were mainly videos and pictorial representations, as indicated by most of the participants.

The themes identified were helpful, understandable, videos, and pictures.

*“Videos were very helpful and understandable.”* **(Participant 3)**

*“Actually, I have seen all your content; ummmm … the photographs of diabetic foot shared, aaa … a fear complex develop, because so far we have neglecting about our foot. After seeing those photos and that, ummm …. actually a fear complex has developed, as a result we have now started taking extra care about our foot.”* **(Participant 5)**

### The usefulness of WhatsApp mode of sharing the educational resources

The WhatsApp mode of sharing the educational resources was useful and appropriate as stated by the study participants. The participants could easily access the resources at their own pace and during their free time.

The themes identified were beneficial, convenient, and accessible.

*“Yes it was very useful, ahhhh … as accessing WhatsApp is easy and um um... unlike other application, in WhatsApp we could open the resources conveniently aaa … at any time we need, when we are free. In other application this is not possible.”* **(Participant 6)**

### Appropriateness of duration of the study, *i.e.*, three months and frequency ( *i.e.*, twice a week on Mondays and Fridays) in which the educational resources were shared

The study duration, *i.e.*, three months, and the frequency ( *i.e.*, twice a week on Mondays and Fridays) in which the educational resources were shared were also mentioned as appropriate by most participants. A few of them, however, found it longer and repetitive.

The themes identified were appropriate, useful, adequate, and repetition.

*“Yes …. appropriate and adequate”* **(Participant 3)**

*“Yes, giving little-little information at a time, um um.. rather than giving whole information, we could ah ah understand. and in our free time we could see the information”* **(Participant 4)**

*“I think it is too long, ah ah … so you could have finished within one or one and half months. And sometimes ummm... the repetition of the same things, I have noticed that, same thing have been repeated in so many WhatsApp messages. It is repetition.”* **(Participant 5)**

### Role of the educational module in improving knowledge and practices of diabetic foot self-management

According to the participants, the educational module effectively enhanced their understanding and implementation of self-management practices for diabetic foot care.

The themes identified were improvement.

*“Definitely, because I was not caring much about my foot but after that um um … I have ahhh..been experiencing some burning sensation in my foot, then uh uh I had that diabetic neuropathy also, I was taking medicine for that also. So my foot is actually a a..red and very thin, and most of the time I was not wearing footwear, I mean uh uh … chappals inside the house, because we are very orthodox people, generally ummm.. don’t wear chappals inside. I was hesitating whether to wear ah ah..chappal inside the house and then finally after reading all your communications aaaa..I felt it better to start using footwear inside the ah ah house also. And regarding that care should be taken about that the uh uh.. foot, I have been seriously following it now. I never used to use this ummm..lotion or ahh.. moisturizer for my foot, but now I have made it a point, aaa.. every night before going to bed, I, I.. will use someee moisturizer and someee lotion and uhhh..taking precautions. And regarding neuropathy, ahhh … actually my family doctor had prescribed ummm.. some medicine and I’m continuously taking it, so it has given me lot of relief.”* **(Participant 5)**

### Limitations of the module

Most interviewees liked the module’s conduct entirely and stated no limitations. A few interviewees, however, mentioned the lack of individualization as a limitation of the IPC module. They added that the module must be tailor-made to meet every individual’s requirements.

The themes identified were lack of individualization/personalization.

*“Ummmm …. Individualization is very very important, not group, that is my suggestion. Each patient is aa having their own problems. But giving mmm same medicine to everybody, aaaa it won’t work. You have to see the patient individually.”* **(Participant 2)**

### Suggestions for further improvement of the educational module

Some of the interviewees also provided suggestions for further improvement of the module. Participants wished to have video conferencing and frequent interactions with the doctors to clarify their concerns. Periodic reviews would be better rather than unnecessary lectures. Further sharing the resources frequently is also stated as helpful by some participants. The rest of them had no further suggestions, as the module in itself was adequate and appropriate.

The themes identified were interaction, follow-up, and frequent.

*“There should be a video conference or ummm interaction and the questions should be ready, ah ah they should straight away ask you questions and a a you should straight away answer it. Or you can tell this session ahh ahh I cannot answer, I will answer in the next session. So that will be ahhh more effective.*” **(Participant 1)**

*“There should be reviews in aaa.. between would be easy rather than ah ah unnecessary lectures, but some may read, I will read at least once, ummm but some will ignore these, aaa rather than these things, on my point of view, ummm.. face to face interactions would be more effective. And aaa..you should aaa… Be factual to the patients, we don’t want to hear your lectures, it should be direct like this aaa the fact, ummm.. what is your prescription or medicine or a a..what you advice. Yaa because we are not coming to hear stories no, it should be factual.*” **(Participant 1)**

*“Yes it can be improved by sharing this information frequently. Ah ah … And very much thank you for all this information.* *”* **(Participant 4)**

## Discussion

An increasing realization highlights the importance of adopting an interdisciplinary, or IP, approach to ensure effective diabetic foot care, emphasizing the requirement for a well-prepared team. ^28^ In response to this requirement, India has introduced a nationwide effort called the Diabetic Foot Education Program (DFEP). This initiative is geared towards enhancing physicians’ knowledge about preventing diabetic foot problems, managing such issues (including debridement and offloading), providing foot-care education, and implementing methods to elevate the quality of healthcare for diabetic foot patients. ^29^

The importance of managing diabetic foot ulcers by an IP diabetic foot ulcer team has been previously mentioned. ^24^^,^^30^ Engaging a multidisciplinary foot care team has demonstrated substantial improvements in reducing amputations and other complications related to DFUs. ^31^^–^^33^

The present study further demonstrates the importance of IP approach to diabetic foot care. In addition to the routine foot care practices, the IP team was also involved in developing and validating the educational module on diabetic foot self-management. This attempt was useful to the study participants, *i.e.*, individuals with diabetes.

Beyond the management of the condition, if the IP team collaboratively engages in raising awareness about the significance of self-care for diabetic feet and advocating the correct techniques for self-managing diabetic foot health, as observed in the current study, it has the potential to reduce the issues linked to diabetic foot complications significantly. It is believed that when different healthcare professionals, *i.e.*, physicians, surgeons, physiotherapists, nurses, dieticians, and podiatrists form a team and constantly reiterate and reinforce the benefits of foot care in diabetes, diabetic foot self-management can be effectively practiced by individuals with diabetes in specific and the community in general.

The constant reinforcement of the knowledge of proper foot care practices in diabetes as provided in the module-led current study was well appreciated by the study participants. It had a substantial positive impact on the participants, enhancing their understanding and implementation of diabetic foot care practices.

Research has uncovered an apparent disparity between the understanding and implementing of foot care practices among people with diabetes. ^1^^,^^2^^,^^15^^–^^18^ This gap needs to be addressed by developing comprehensive behavior change strategies. Implementing comprehensive risk assessments for diabetic foot complications and foot care based on prevention, education, and support at the community level has become extremely important. ^15^ Awareness sessions on diabetic foot self-management that are readily available to the community can help address this issue.

In the present study, awareness was created through an educational module through the most commonly accessed platform, *i.e.*, WhatsApp. The WhatsApp mode of sharing the educational resources was useful and appropriate as stated by the study participants. The educational resources were mainly focused on diabetes and its health implications, diabetic foot and self-management, and the importance of interprofessional education in diabetic foot care. Participants could easily access the resources at their own pace and during their free time. This attempt made accessibility easier. It also successfully changed the participants’ behavior toward proper foot care practices.

The American Diabetes Association advocates for the utilization of initiatives like “Diabetes Self-Management Education” (DSME) to enhance understanding, behavior, and self-care, particularly when it comes to foot care for individuals dealing with diabetes. ^34^ These programs have the potential to avert and address complications associated with diabetes, enhance the quality of life for patients, and alleviate the economic impact. ^34^^,^^35^ Similar observations were made in the current study.

Numerous prior studies have centered on assessing the influence of educational interventions on enhancing the knowledge and attitudes of individuals with diabetes regarding diabetic foot care.

A straightforward in-person education approach proved to be successful in elevating awareness about foot care, and it demonstrated the potential to enhance motivation and induce behavioral changes among individuals with Type II Diabetes Mellitus. ^36^ In the present study some of the participants preferred face-to-face interactions over online interactions. But this approach will be suitable during stand-alone sessions, *i.e.*, a one-day workshop/session, rather than the implementation of a longer-term educational module, *i.e.*, three months in this case. Further, the face-to-face approach may not be suitable while considering many participants, wherein the issues of lack of attendance or participation may surface as the module/program progresses. Therefore, online interactions are preferable. Measures should be taken to make online interactions more participatory to enhance participant satisfaction and learning.

The efficiency of a culturally customized self-management education (SME) program for foot care was previously examined. The program encompassed an engaging group discussion that employed a narrative video, a PowerPoint presentation, and a printed guide. Following the intervention, foot care practices had a noticeable enhancement, as indicated by the results. ^19^

These sessions were, however, standalone sessions conducted at a single point in time, unlike the prolonged three-month-long educational module described in the current study.

The influence of an intensive diabetes foot education initiative aimed at veterans at elevated risk of foot ulcers was previously assessed. This education program enhanced the knowledge and practices related to foot care among high-risk patients. However, the study emphasized the need to explore methods to improve the accessibility of these educational sessions. ^37^

Providing caregivers with ongoing interdisciplinary education on foot care has bolstered the family support system for managing diabetic ulcers. ^38^ The present study agrees with the same and adds that ongoing IP education (in the form of modules) on the proper foot care practices can be more effective in instilling a behavioral change among individuals with diabetes and their caregivers.

While prior research has underscored the benefits of educational interventions for enhancing understanding and practices related to diabetic foot care, the potential of IP collaborations in diabetic foot self-management remains relatively underexplored. The present study has attempted to explore the same. An IP approach to diabetic foot education helps to address the components holistically. Individuals with diabetes are comprehensively provided with all the relevant information using an IP approach.

An extensive review of the available literature uncovered limited evidence supporting the efficacy of educational interventions for enhancing diabetic foot management practices within the unique context of India. India, known for its rich cultural, ethnic, and economic diversity, employs various approaches to managing diabetes and its related complications, including diabetic foot issues. Limited access to healthcare services due to financial constraints and geographical barriers exacerbates the problem of inadequate foot care practices among individuals with diabetes. Consequently, there is a pressing need to develop accessible awareness sessions or modules tailored for these underserved populations to enhance knowledge and promote better self-management practices for diabetic foot care.

The creation of an IPC educational module and the evaluation of its impact on enhancing knowledge and practices associated with diabetic foot self-management, even before diabetic foot ulcers develop, has been a rarely explored topic. Consequently, this current study seeks to address this gap. It demonstrates the effectiveness of the IP collaborative educational module in enhancing knowledge and practices related to foot care in individuals with diabetes. Extending the module’s duration, as in this case, to three months, along with ongoing follow-up, can significantly boost its efficacy in improving foot care knowledge and practices, thus helping prevent complications linked to diabetic foot issues. Easy accessibility to the educational program/module provided through WhatsApp was also deeply appreciated by the study participants.

Equipping the patient with insights into potential complications and stressing the significance of proper medical care can reduce the likelihood of complications and enhance adherence to diabetic foot care treatment. ^39^ The current study demonstrates that an IPC educational module can effectively address these aspects.

### Limitations and future recommendations

Although the implementation of the educational module was well appreciated by the study participants, a few concerns were raised. Some participants favored the repetitive sharing of the information, stating that it aided in changing their attitude towards following proper foot care practices. On the contrary, a few others found the process tedious and the duration unnecessarily prolonged. Measures need to be taken to address these issues. Suggestions to customize the educational module based on the individual’s needs were also expressed. More face-to-face interactions and factual communications were also preferred. The module can be further improved based on these suggestions before future implementation to enhance its effectiveness and acceptance.

The current study was piloted with a small cohort of participants, and only seven participants consented to a telephonic interview. It calls for further refinement in the execution of the educational module so that it can be seamlessly accessible to a large group of participants, *i.e.*, individuals with diabetes. The issues of participants’ fidelity/adherence to the educational module during the course of the intervention should also be adequately explored and suitable measures should be taken to increase their consistency in learning/following the programs.

Considering the issues of creating health inequality, the mode of delivery of the educational module, *i.e.*, online via WhatsApp is not scalable considering individuals who do not have access to WhatsApp-supported devices. Thus, the findings cannot be generalizable. One possible solution to address this issue could be involving the care givers, next to kin or family members of the individuals with diabetes who have access to WhatsApp-supported devices to inform and educate them.

## Conclusion

The IPC Educational Module can be useful in enhancing the knowledge and practices of self-managing diabetic foot care among individuals with Type II Diabetes Mellitus. The study participants express that the module has value and exhibits usefulness in improving their understanding and implementation of diabetic foot self-management. The method of delivery, as well as the learning materials shared, piqued their interest in acquiring knowledge about proper foot care practices. Leveraging WhatsApp to access educational resources was considered “beneficial” and highly “convenient”.

Furthermore, by personalizing the module to cater to individual needs, this educational approach could enhance foot care knowledge and practices in individuals with diabetes. Consistent follow-ups and ongoing reminders regarding foot care can foster a shift in attitude, consequently leading to improved healthcare practices among individuals with diabetes. In the long term, this can decrease hospital admissions for diabetic foot-related complaints, fewer amputations and mortality rates among diabetic foot patients, and an overall enhancement in quality of life for both individuals and society.

## Data Availability

Figshare: Development and Validation of an Interprofessional Collaborative Educational Module on the Self-Management of Foot for Individuals with Type II Diabetes Mellitus. https://doi.org/10.6084/m9.figshare.24947508. ^40^ The project contains the following underlying data:
- Evaluation tool_educational module.xlsx - Evaluation tool_educational resources.xlsx - WhatsApp Chat with Diabetic foot care.txt - Interview transcript 1.docx - Interview transcript 2.docx - Interview transcript 3.docx - Interview transcript 4.docx - Interview transcript 5.docx - Interview transcript 6.docx - Interview transcript 7.docx Figshare: Development and Validation of an Interprofessional Collaborative Educational Module on the Self-Management of Foot for Individuals with Type II Diabetes Mellitus. https://doi.org/10.6084/m9.figshare.24947508. ^40^ The project contains the following extended data:
- Annexure 1_Validation tool for the educational module and resources.docx.pdf - Annexure 2_Interview guide.pdf - Annexure 3_Educational module_self management of diabetic foot.pdf - Annexure 4_Educational Resources.pdf - Annexure 5_Participant responses and Themes.pdf - Annexures.zip TIDieR (Template for Intervention Description and Replication); COREQ (COnsolidated criteria for REporting Qualitative research) Checklist; and CONSORT (Consolidated Standards of Reporting Trials). Checklist for “Development and validation of an interprofessional collaborative educational module on the self-management of foot for individuals with Type II Diabetes Mellitus” https://doi.org/10.6084/m9.figshare.24947508. ^40^ Data are available under the terms of the Creative Commons Attribution 4.0 International license (CC-BY 4.0).
