# Supplementary material for: Development and validation of an interprofessional collaborative educational module on the self-management of foot for individuals with type II diabetes mellitus in India
Source: F1000Res. 2025 Feb 4;13:386. Originally published 2024 Apr 25. [Version 4] doi: 10.12688/f1000research.146943.4 (PMC12254720; doi:10.12688/f1000research.146943.4)
Supplement: Peer review history

# Reviewer report: Reviewer response for version 3

**DOI:** 10.5256/f1000research.176694.r358162

**Published:** 2025-01-23

**Authors:** Anas Ababneh

**Affiliations:** 1 Yarmouk University, Irbid, Jordan

The authors have done a great job in improving this manuscript. I have one comment:

 There is no need for this statement in the conclusion section as we have no idea yet about the effectiveness of the suggested IPC in preventing DFUs:

 "By promoting collaborative care approaches, the IP educational module can contribute to better preventive practices, potentially reducing the risk of complications like foot ulcers and amputations in individuals with diabetes"

Is the work clearly and accurately presented and does it cite the current literature?

Yes

If applicable, is the statistical analysis and its interpretation appropriate?

Partly

Are all the source data underlying the results available to ensure full reproducibility?

Yes

Is the study design appropriate and is the work technically sound?

Yes

Are the conclusions drawn adequately supported by the results?

Partly

Are sufficient details of methods and analysis provided to allow replication by others?

Partly

Reviewer Expertise:

Diabetic foot

I confirm that I have read this submission and believe that I have an appropriate level of expertise to confirm that it is of an acceptable scientific standard.

# Response

**Published:** 2025-01-28

**Authors:** Sushma Prabhath

**Affiliations:** Anatomy, Manipal Academy of Higher Education, Manipal, Karnataka, India

Dear Reviewer

 Many thanks for your supportive comments. We have made the necessary changes in the conclusion section as advised.

# Reviewer report: Reviewer response for version 2

**DOI:** 10.5256/f1000research.170241.r327304

**Published:** 2024-10-11

**Authors:** Anas Ababneh

**Affiliations:** 1 Yarmouk University, Irbid, Jordan

This study aimed to provide a validated educational program about foot self-care for people with diabetes.

 Comments:

- The conclusion in the abstract is not appropriate as this study did not test the effectiveness of the suggested educational program on adherence outcome. It is just suggesting a validated educational content.
- The introduction suggest the importance of creating foot self-management educational intervention in India while the study title and abstract does not reflect this. Please match!
- Could you add details about how the educational resources were developed from reviewing the literature.
- Did the translation of the educational material considered cultural appropriateness?
- I suggest to offer the educational module as (Additional file)
- The pilot testing looks like investigate participants’’ experience with the educational intervention through collecting some thoughts as qualitative data. I am not sure if checking the effectiveness of the educational module is suitable in this context.
- I wonder as many people (internal and external experts) participated in validating the educational module, why not the results are expressed as content validity index instead of just presenting the mean of rating score?
- I am not sure if the conclusion section reflect the aim of the article. I expected to see the main findings of the Delphi process i.,e validated educational module and pilot testing indicating  good experience by participants etc. The provide evidence in this work is far to be substantial  to conclude that IPC educational model is useful in enhancing knowledge or improving adherence to foot self-care as mentioned in the abstract. Please re-write conclusion concisely to tell the reader about how much the tested educational model is valid and feasible. Future research can be recommended to test its effectiveness.

Is the work clearly and accurately presented and does it cite the current literature?

Yes

If applicable, is the statistical analysis and its interpretation appropriate?

Partly

Are all the source data underlying the results available to ensure full reproducibility?

Yes

Is the study design appropriate and is the work technically sound?

Yes

Are the conclusions drawn adequately supported by the results?

Partly

Are sufficient details of methods and analysis provided to allow replication by others?

Partly

Reviewer Expertise:

Diabetic foot

I confirm that I have read this submission and believe that I have an appropriate level of expertise to confirm that it is of an acceptable scientific standard, however I have significant reservations, as outlined above.

# Response

**Published:** 2025-01-07

**Authors:** Sushma Prabhath

**Affiliations:** Anatomy, Manipal Academy of Higher Education, Manipal, Karnataka, India

Dear Reviewer

 Many thanks for reviewing our manuscript and providing your positive insights into its improvements. Your valuable suggestions have thoroughly supported us in refining the manuscript.

**Reviewer’s overview:** This study aimed to provide a validated educational program about foot self-care for people with diabetes.

**Response to comment:** Thank you for your support. We value your suggestions and have made all the necessary corrections and inclusions as advised.

**A point-to-point reply to the stated comments is mentioned below:**

**Comments/ Suggestions**

**Clarifications/Modifications**

**1**

***Abstract***

**Comment: **The conclusion in the abstract is not appropriate as this study did not test the effectiveness of the suggested educational program on adherence outcome. It is just suggesting validated educational content.

**Response: **We agree with the reviewer’s suggestion. Hence the conclusion is now corrected highlighting the development and validation of the educational module and its importance.

**2**

**Comment: **The introduction suggest the importance of creating foot self-management educational intervention in India while the study title and abstract does not reflect this. Please match!

**Response: **The importance of creating foot self-management educational intervention in India is now reflected in the study title and abstract as advised.

**3**

**Comment: **Could you add details about how the educational resources were developed from reviewing the literature.

**Response: **The process of developing the educational resources from reviewing the literature is detailed as advised in the methodology section as advised.

**4**

**Comment: **Did the translation of the educational material considered cultural appropriateness?

**Response: **Since the study was carried out in the Indian population, the translation of the educational material was carried out considering the cultural appropriateness.

 A statement of effect is included in the methodology section of the manuscript

**5**

**Comment: **I suggest to offer the educational module as (Additional file)

**Response: **The educational module along with the resources are available as Annexures 3 and 4 in the extended data. A statement of effect is mentioned in the ‘results’ section of the manuscript.

**6**

**Comment: **The pilot testing looks like investigate participants’’ experience with the educational intervention through collecting some thoughts as qualitative data. I am not sure if checking the effectiveness of the educational module is suitable in this context.

**Response: **We agree with the reviewers comments. Hence the word ‘effectiveness’ is being replaced by the term ‘usefulness’ wherever applicable, thereby fitting the context of the current study.

**7**

**Comment: **I wonder as many people (internal and external experts) participated in validating the educational module, why not the results are expressed as content validity index instead of just presenting the mean of rating score?

**Response: **The validation of the education of the educational module was carried out using the following reference article as the basis. Hence Content validity index was not considered.

 Source: Ribeiro ZMT, Spadella MA: Content validation of educational material on healthy eating for children under two years of age. Rev. Paul. Pediatr. 2018 Apr-Jun;36(2):155–163. 29412433 10.1590/1984-0462/;2018;36;2;00007 PMC6038784

**8**

**Comment: **I am not sure if the conclusion section reflect the aim of the article. I expected to see the main findings of the Delphi process i.,e validated educational module and pilot testing indicating  good experience by participants etc. The provide evidence in this work is far to be substantial  to conclude that IPC educational model is useful in enhancing knowledge or improving adherence to foot self-care as mentioned in the abstract. Please re-write conclusion concisely to tell the reader about how much the tested educational model is valid and feasible. Future research can be recommended to test its effectiveness.

**Response: **The conclusion section is rewritten as advised considering the points raised by the reviewer. The now reflects the aim of the article.

# Reviewer report: Reviewer response for version 1

**DOI:** 10.5256/f1000research.161075.r302217

**Published:** 2024-08-09

**Authors:** Joseph Ngmenesegre Suglo

**Affiliations:** 1 Kings College London, London, UK

The authors presented a paper on an interesting and clinically relevant topic “Development and validation of an interprofessional collaborative educational module on the self-management of foot for individuals with type II diabetes mellitus” They developed and validated an interprofessional collaborative (IPC) educational program involving HCPs to efficiently as a way of promoting healthy footcare practices. The paper however will require major revisions before it is accepted for indexing.

 Abstract

 The writing style is difficult to read in places and I feel that the whole paper could do with a review by a copy editor.

 The author should be consistent in the use of Type II or type 2 diabetes. Use one throughout the manuscript.

 The first sentence within the methods section of the abstract not required. This first line should be used to state explicitly the study design used. Avoid repetitions, the study aim has been repeated in different forms. It is seen in the background and under methods again.

 Introduction

 Many statements/paragraphs within the manuscript require references eg The worldwide “occurrence of DFUs varies between 5% and 7.5% among individuals with diabetic neuropathy” (REF required).

 Design of the study:

 The reporting guidelines used should be stated and cited within the methods sections of the study. I have seen the CONSORT guidelines checklist for randomised trials completed and uploaded – I am not sure if this is the appropriate guideline for this study as the study contains more qualitative elements than a randomised trial. The authors may want to assess other reporting guidelines such as the CORECQ guidelines for their suitability.

 The study seems to use sequential multimethod to develop and evaluate the module. It is quite difficult to understand exact design used in this study. If possible, can the authors give a summary of the design/methods using a flow diagram or framework? Also be very coherent in presenting the various phases of the study, clearly indicating how each phase informed the next phase.

 How were members of the FGD identified and by who? What criteria was use in recruiting them? Similarly, persons who carried out the validation of the module? How, and who recruited them?

 Participants that were interviewed, what sampling approach was used?

 The educational resources shared on whatsapp, who shared this information? Was it sent to participants on their individual phones or they were in single whatsapp group to receive the information? How did the authors assess whether participants actually read or watched the resources shared on whatsapp? This relates to participants fidelity/adherence to the intervention?

 How about internet accessibility for whatsapp users? Did you provide internet data bundle or incentive to participants?

 “The educational resources were developed in English and the local language, *i.e.*, Kannada” – Was it first developed in English and translated or the vise versa? what methods were used to ensure consistency and accuracy?

 Discussion

 Thinking about creating health inequality – what happens to persons with diabetes who have no access to whatsapp supported devices? Is this mode of delivery scalable? Will the findings be generalizable? Discuss these as part of the study limitations.

 “A straightforward in-person education approach proved to be successful in elevating awareness about foot care, and it demonstrated the potential to enhance motivation and induce behavioral changes among individuals with type 2 diabetes. ^35^ The present study has identified the same as its limitations. The participants preferred face-to-face interaction rather than receiving the resources online. This approach may, however, not be suitable while addressing a large cohort of participants and needs to be suitably managed.”  - I am not sure the message being communicated here – Do you mean participants in your study preferred face-to-face to online?

 The paper will benefit from proof reading to correct avoidable mistakes eg improvised instead of improved as stated under discussion. – “The module can be further improvised based on these suggestions before future implementation to enhance its effectiveness and acceptance”

 Conclusion

 The authors stated that “The IPC Educational Module proves highly effective in enhancing the knowledge and practices of self-managing diabetic foot care among individuals with Type II Diabetes mellitus” – I wish they don’t have to use the word “effective” which carries a statistical connotation. However, inferential statistics were not part of this study to evaluate participants' knowledge and practices. This was rather done quantitatively.

Is the work clearly and accurately presented and does it cite the current literature?

Partly

If applicable, is the statistical analysis and its interpretation appropriate?

Yes

Are all the source data underlying the results available to ensure full reproducibility?

Yes

Is the study design appropriate and is the work technically sound?

Partly

Are the conclusions drawn adequately supported by the results?

Partly

Are sufficient details of methods and analysis provided to allow replication by others?

Partly

Reviewer Expertise:

Diabetes care modules

I confirm that I have read this submission and believe that I have an appropriate level of expertise to confirm that it is of an acceptable scientific standard, however I have significant reservations, as outlined above.

# Response

**Published:** 2024-08-26

**Authors:** Sushma Prabhath

**Affiliations:** Anatomy, Manipal Academy of Higher Education, Manipal, Karnataka, India

Dear Reviewer,

 Many thanks for reviewing our manuscript and providing your positive insights towards its improvements. Your valuable suggestions have thoroughly supported us in refining the manuscript.

**Reviewer’s overview:** The authors presented a paper on an interesting and clinically relevant topic “Development and validation of an interprofessional collaborative educational module on the self-management of foot for individuals with type II diabetes mellitus” They developed and validated an interprofessional collaborative (IPC) educational program involving HCPs to efficiently as a way of promoting healthy footcare practices. The paper however will require major revisions before it is accepted for indexing.

**Response to comment:** We value your suggestions and have made all the necessary corrections and inclusions as advised. As stated, the manuscript is also language edited to enhance its readability.

**A point-to-point reply to the stated comments is mentioned below:**

**Comments/ Suggestions**

**Clarifications/Modifications**

**1.** ***Abstract***

 The writing style is difficult to read in places and I feel that the whole paper could do with a review by a copy editor.

**Response to comment: **The manuscript has been subjected to language editing to enhance its readability

**2. **The author should be consistent in the use of Type II or type 2 diabetes. Use one throughout the manuscript.

**Response to comment: **We have now maintained the consistency of using ‘Type II Diabetes Mellitus’ throughout the manuscript as advised.

**3. **The first sentence within the methods section of the abstract not required. This first line should be used to state explicitly the study design used. Avoid repetitions, the study aim has been repeated in different forms. It is seen in the background and under methods again.

**Response to comment: **The necessary corrections are made as suggested. The first line under methods section in the abstract states about the development of the educational module followed by details of its validation. The objective of the module previously mentioned in the methods is now removed to avoid repetition (as previously mentioned as the aim of the study in the ‘Background’)

**4.** ***Introduction***

 Many statements/paragraphs within the manuscript require references eg The worldwide “occurrence of DFUs varies between 5% and 7.5% among individuals with diabetic neuropathy” (REF required).

**Response to comment: **The references are adequately provided as advised. The statements/paragraphs requiring referencing are suitable cited.

 Suitable changes are made in the reference list too.

**5.** ***Design of the study:***

 The reporting guidelines used should be stated and cited within the methods sections of the study. I have seen the CONSORT guidelines checklist for randomised trials completed and uploaded – I am not sure if this is the appropriate guideline for this study as the study contains more qualitative elements than a randomised trial. The authors may want to assess other reporting guidelines such as the CORECQ guidelines for their suitability.

**Response to comment: **The reporting guidelines are stated and cited appropriately within the methods section as advised. We have included the following (as found appropriate): TIDieR (Template for Intervention Description and Replication); COREQ (COnsolidated criteria for REporting Qualitative research) Checklist and CONSORT (Consolidated Standards of Reporting Trials).

 TIDieR checklist for reporting the description of the educational intervention and its replication

 COREQ checklist for reporting the qualitative research carried out

 CONSORT checklist for reporting the usefulness of the educational intervention carried out as a clinical trial on the study participants, i.e., individuals with diabetes.

**6. **The study seems to use sequential multimethod to develop and evaluate the module. It is quite difficult to understand the exact design used in this study. If possible, can the authors give a summary of the design/methods using a flow diagram or framework? Also be very coherent in presenting the various phases of the study, clearly indicating how each phase informed the next phase.

**Response to comment: **A flowchart summarizing the methodology is now presented as figure 1. It clearly indicates how each phase leads to the next. Also a detailed explanation is provided under each phase/step of the methodology.

**7. **How were members of the FGD identified and by who? What criteria was use in recruiting them? Similarly, persons who carried out the validation of the module? How, and who recruited them?

**Response to comment: **The principal investigator (first author of the study) identified and invited the following HCPs, i.e., general physician, an endocrinologist, a surgeon, a physiotherapist, a dietician, and a nursing professional to constitute an IP team. The IP team also included an anatomist experienced in health professions education. All the HCPs invited to form the IP team were involved in diabetic care of the patients in their respective workplace.

 They were the members who were involved in the group discussion that aimed at the development of the educational module. 

 The individuals who carried out the validation of the educational module were a team of both external and internal experts. The internal experts were the same HCPs who constituted the IP team and were previously involved in the FGD leading to the development of the educational module.

 The external experts involved in the validation of the educational were other HCPs cum experts in the field of medical education not related to the study. They were not involved in the constitution of IP team and the development process of the educational module.

 The current study is a part of a larger project designed to develop an interprofessional educational module to educate individuals with diabetes about proper practices of diabetic foot care. Relevant reference indicating the same is already included (cited-reference number 24) in the manuscript under “Development of the educational module”. However, to provide better clarity the sentences have been reframed as per reviewer’s comment.

 Relevant statements of effect have been included in the manuscript wherever appropriate.

**8. **Participants that were interviewed, what sampling approach was used?

**Response to comment:** *Convenient sampling was used. At the end of the implementation of the education module, i.e., sharing of the educational resources in the WhatsApp group after three months, the Participants were asked to express their willingness related to attending a telephonic interview related to the usefulness of the session. The consenting participants were telephonically approached for the interview.*

 Statements of this effect are included in the manuscript.

**9. **The educational resources shared on whatsapp, who shared this information? Was it sent to participants on their individual phones or they were in single whatsapp group to receive the information? How did the authors assess whether participants actually read or watched the resources shared on whatsapp? This relates to participants fidelity/adherence to the intervention?

 How about internet accessibility for whatsapp users? Did you provide internet data bundle or incentive to participants?

**Response to comment:** *The principal investigator created a WhatsApp group for the study participants and the educational resources were shared therein. The information regarding creating the WhatsApp group and sharing of educational resources was previously conveyed to the participants during the recruitment process. The participants were encouraged to regularly review the resources. To ensure whether participants read or watched the resources shared on WhatsApp, the number of views per entry was reviewed and tracked. No other measures were taken to ensure participants’ fidelity/adherence to the intervention.*

*The users (participants) used their own internet data pack to access WhatsApp. No internet data bundle facilities or incentives were provided to the participants for the same by the researchers.*

 Statements of this effect are included in the methodology section of the manuscript.

 Issues related to participants fidelity/adherence to the intervention are also mentioned in the study limitations. 

**10. **“The educational resources were developed in English and the local language, *i.e.*, Kannada” – Was it first developed in English and translated or the vise versa? what methods were used to ensure consistency and accuracy?

**Response to comment: **Yes, *the educational resources were first developed in English and later translated to the local language, i.e., Kannada.*

*To ensure consistency and accuracy of translated content, the following measures were followed:*

*Back-Translation: After translating the content from English to the local language, i.e., Kannada, we had a different translator (who was not involved in the initial translation) translate the content back into English. It ensured that the translated version’s accuracy and consistency reflected the original content.*

*Bilingual Expert Review: We also invited bilingual experts (fluent in both English and Kannada) to review the translated content. The experts assessed the accuracy of the translation, ensuring that it captured both the technical and cultural nuances of the original material.*

 Statements of this effect are included in the methodology section of the manuscript.

**11.** ***Discussion***

 Thinking about creating health inequality – what happens to persons with diabetes who have no access to whatsapp supported devices? Is this mode of delivery scalable? Will the findings be generalizable? Discuss these as part of the study limitations.

**Response to comment: **We value the reviewer’s suggestions.

 However, in the present study, the inclusion criteria for recruiting participants (individuals with diabetes) clearly states that “The participants comfortable in using the “ WhatsApp”, i.e. a social media tool were preferred”

 Considering the issues on creating health inequality, we totally agree that the *mode of delivery of the educational module, i.e., online via WhatsApp is not scalable considering individuals who have no access to WhatsApp supported devices. Thus, the findings cannot be generalizable. One possible solution to address this issue could be involving the care givers, next to kin or family members  of the individuals with diabetes who have access to WhatsApp supported devices to inform and educate them.*

 Statements of this effect is included in the ‘study limitations’ of the manuscript to provide clarity as advised.

**12. **“A straightforward in-person education approach proved to be successful in elevating awareness about foot care, and it demonstrated the potential to enhance motivation and induce behavioral changes among individuals with type 2 diabetes. ^35^ The present study has identified the same as its limitations. The participants preferred face-to-face interaction rather than receiving the resources online. This approach may, however, not be suitable while addressing a large cohort of participants and needs to be suitably managed.”  - I am not sure the message being communicated here – Do you mean participants in your study preferred face-to-face to online?

**Response to comment: **Yes, some of the participants in our study preferred face-to-face in comparison to online interactions. But this approach will be suitable during stand-alone sessions, i.e., a one-day workshop/session and not during the implementation of an educational module of longer duration, i.e., three-months in this case. Further, the face-to face approach may not be suitable while considering many participants, wherein the issues of lack of attendance or participation may surface as the module/program progresses. Therefore, online mode of interactions is preferable. Measures should be taken to make the online interactions more participatory to enhance participant satisfaction and learning.

 Statements of this effect is included in the discussion section of the manuscript to provide more clarity

**13. **The paper will benefit from proof reading to correct avoidable mistakes eg improvised instead of improved as stated under discussion. – “The module can be further improvised based on these suggestions before future implementation to enhance its effectiveness and acceptance”

**Response to comment: **The necessary corrections are made as suggested. A thorough proof reading of the manuscript is carried out to correct the mistakes. Further, the manuscript is also language edited to enhance its readability.

**14.** ***Conclusion***

 The authors stated that “The IPC Educational Module proves highly effective in enhancing the knowledge and practices of self-managing diabetic foot care among individuals with Type II Diabetes mellitus” – I wish they don’t have to use the word “effective” which carries a statistical connotation. However, inferential statistics were not part of this study to evaluate participants' knowledge and practices. This was rather done quantitatively.

**Response to comment: **We value the reviewer’s suggestion. Since inferential statistics were not part of the study to evaluate the participants knowledge and practices and the usefulness of the module was assessed only qualitatively (using personal interviews with the study participants); we have therefore replaced the statement  “proves highly effective…..”. It now reads as follows: The IPC Educational Module **can be useful **in enhancing the knowledge and practices of self-managing diabetic foot care among individuals with Type II Diabetes mellitus. The study participants express that the module has value and exhibits **usefulness** in improving their understanding and implementation of diabetic foot self-management.

# Reviewer report: Reviewer response for version 1

**DOI:** 10.5256/f1000research.161075.r279959

**Published:** 2024-05-24

**Authors:** Suyog Sindhu

**Affiliations:** 1 Pharmacology, King George's Medical University, Lucknow, Uttar Pradesh, India

**General Overview:**

 The presented study addresses a significant issue in diabetic care— the management of diabetic foot health through interprofessional collaboration. The paper details the development and validation of an educational module aimed at enhancing self-management practices among individuals with type 2 diabetes mellitus. The study employs a robust methodological framework and provides evidence supporting the efficacy of the educational intervention.

**Detailed Review:**

**Clarity and Accuracy of Presentation and Literature Cited:**

 The manuscript is clearly and accurately presented, with well-organized sections that facilitate understanding.

 The background section effectively highlights the importance of foot self-care in diabetes management and the need for an interprofessional approach.

 Relevant literature is cited appropriately, grounding the study within the context of existing research on diabetic foot care and interprofessional education.

**Study Design Appropriateness and Technical Soundness:**

 The study design is appropriate for the research objectives. The use of the Delphi method for module validation ensures expert consensus, lending credibility to the educational content.

 The implementation of a three-month-long module with structured sessions is a well-thought-out approach, aligning with educational best practices.

 The use of diverse educational materials (pamphlets, flowcharts, handouts, case-based cartoons, and videos) caters to varied learning preferences and enhances engagement.

**Detailing of Methods and Analysis for Replication:**

 The manuscript provides sufficient details regarding the development of the educational module, the Delphi validation process, and the subsequent testing phase.

 The description of the two-stage validation process, including the expert panel composition and criteria for consensus, is comprehensive.

 Details on the module's content delivery via Whatsapp and the types of educational resources shared are clearly articulated.

 The methods section enables replication by other researchers, ensuring the study's reproducibility.

**Statistical Analysis and Interpretation:**

 The study utilizes qualitative analysis techniques, including conversation analysis and in-depth interviews, to evaluate the module's efficacy.

 The interpretation of the qualitative data is appropriate, highlighting the participants' improved knowledge and practices in diabetic foot care.

 While the study lacks quantitative analysis, the qualitative insights provide valuable evidence supporting the module's effectiveness.

**Availability of Source Data for Reproducibility:**

 The manuscript mentions that all participating experts consensually validated the module, indicating transparency in the validation process.

 Detailed raw data from the in-depth interviews and conversation analysis has been provided.

**Support of Conclusions by Results:**

 The conclusions drawn are well-supported by the results. The consensus validation by experts and the positive feedback from participants underscore the module's effectiveness.

 The study convincingly argues that the IPC educational module can improve adherence to proper diabetic foot care practices, potentially reducing foot ulcers and amputations.

 The findings align with the stated objectives and contribute valuable insights into the role of interprofessional education in diabetic care.

**Recommendations:**

 Quantitative Evaluation: Consider incorporating quantitative measures, such as pre- and post-intervention assessments, to complement the qualitative findings and provide a more robust evaluation of the module's impact.

 Extended Follow-up: Conduct a longer follow-up period to assess the sustained impact of the educational module on participants' foot care practices and health outcomes.

**Conclusion:**

 The study presents a well-designed and thoroughly validated educational module that demonstrates significant potential in improving diabetic foot care through interprofessional collaboration. The manuscript is technically sound, methodologically robust, and offers valuable contributions to the field of diabetic education and care.

Is the work clearly and accurately presented and does it cite the current literature?

Yes

If applicable, is the statistical analysis and its interpretation appropriate?

Yes

Are all the source data underlying the results available to ensure full reproducibility?

Yes

Is the study design appropriate and is the work technically sound?

Yes

Are the conclusions drawn adequately supported by the results?

Yes

Are sufficient details of methods and analysis provided to allow replication by others?

Yes

Reviewer Expertise:

medical education, interprofessional education and clinical pharmacology

I confirm that I have read this submission and believe that I have an appropriate level of expertise to confirm that it is of an acceptable scientific standard.

# Response

**Published:** 2024-07-10

**Authors:** Sushma Prabhath

**Affiliations:** Anatomy, Manipal Academy of Higher Education, Manipal, Karnataka, India

Dear Reviewer (Dr. Suyog Sindhu)

 Many thanks for your supporting lines on reviewing comments to the manuscript. It was a pleasure to read through your insights. We value the positive and appreciative lines provided by you.

 About the recommendations provided by you, your suggestions are well taken and we are planning to scale up the current study to a larger population (in the near future) wherein the points provided shall be duly considered in our future steps:

**Recommendations:**

 1. Quantitative Evaluation: Consider incorporating quantitative measures, such as pre- and post-intervention assessments, to complement the qualitative findings and provide a more robust evaluation of the module's impact.

**Response to reviewer's comments: **We value your suggestion and shall definitely consider incorporating quantitative measures in our future attempts (future-ongoing research) complementing the qualitative findings to provide a more robust evaluation of the module's impact. Based on the current study findings, we are also aiming to refine and redefine the module to meet the requirements of the diverse population (i.e., individuals with diabetes).

 2. Extended Follow-up: Conduct a longer follow-up period to assess the sustained impact of the educational module on participants' foot care practices and health outcomes.

**Response to reviewer's comments: **Your suggestion is well taken and we shall aim to follow the same in our future attempts.
